# Dispersal patterns and population genetic structure of Aedes albopictus (Diptera: Culicidae) in three different climatic regions of China

**DOI:** 10.1186/s13071-020-04521-4

**Published:** 2021-01-06

**Authors:** Jian Gao, Heng-Duan Zhang, Xiao-Xia Guo, Dan Xing, Yan-De Dong, Ce-Jie Lan, Ge Wang, Chao-Jie Li, Chun-Xiao Li, Tong-Yan Zhao

**Affiliations:** State Key Laboratory of Pathogen and Biosecurity, Beijing Institute of Microbiology and Epidemiology, Beijing, 100071 China

**Keywords:** *Aedes albopictus*, Genetic variation, Haplotype, Dispersion pattern, Climatic regions, Microsatellite loci, Environmental factors

## Abstract

**Background:**

*Aedes albopictus* is an indigenous primary vector for dengue and Zika viruses in China. Compared with its insecticide resistance, biology and vector competence, little is known about its genetic variation, which corresponds to environmental variations. Thus, the present study examines how *Ae. albopictus* varies among different climatic regions in China and deciphers its potential dispersal patterns.

**Methods:**

The genetic variation and population structure of 17 *Ae. albopictus* populations collected from three climatic regions of China were investigated with 11 microsatellite loci and the mitochondrial *coxI* gene.

**Results:**

Of 44 isolated microsatellite markers, 11 pairs were chosen for genotyping analysis and had an average PIC value of 0.713, representing high polymorphism. The number of alleles was high in each population, with the *n*_*e*_ value increasing from the temperate region (3.876) to the tropical region (4.144). Twenty-five *coxI* haplotypes were detected, and the highest diversity was observed in the tropical region. The mean Ho value (ca. 0.557) of all the regions was significantly lower than the mean He value (ca. 0.684), with nearly all populations significantly departing from HWE and displaying significant population expansion (*p* value < 0.05). Two genetically isolated groups and three haplotype clades were evaluated via STRUCTURE and haplotype phylogenetic analyses, and the tropical populations were significantly isolated from those in the other regions. Most genetic variation in *Ae. albopictus* was detected within populations and individuals at 31.40 and 63.04%, respectively, via the AMOVA test, and a relatively significant positive correlation was observed among only the temperate populations via IBD analysis (*R*^2^ = 0.6614, *p* = 0.048). Recent dispersions were observed among different *Ae. albopictus* populations, and four major migration trends with high gene flow (Nm > 0.4) were reconstructed between the tropical region and the other two regions. Environmental factors, especially temperature and rainfall, may be the leading causes of genetic diversity in different climatic regions.

**Conclusions:**

Continuous dispersion contributes to the genetic communication of *Ae. albopictus* populations across different climatic regions, and environmental factors, especially temperature and rainfall, may be the leading causes of genetic variation.

**Graphical abstract:**

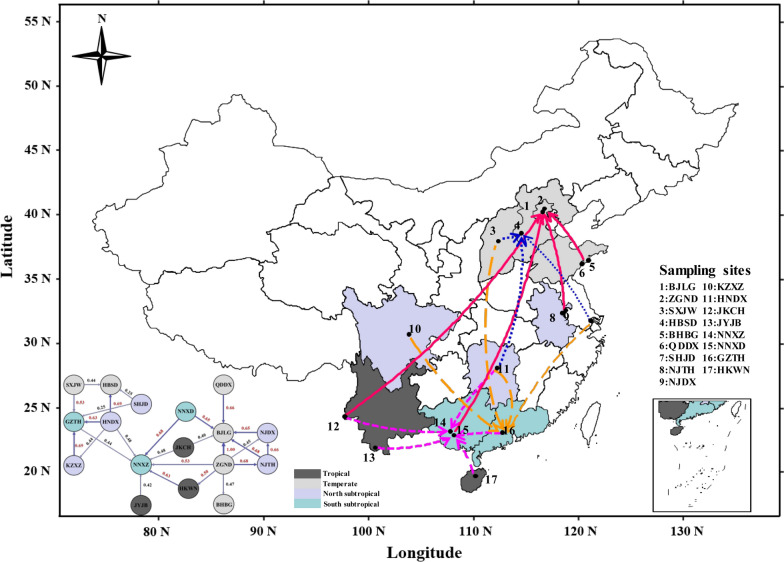

## Background

*Aedes (Stegomyia) albopictus*, also known as the Asian tiger mosquito, is an epidemiologically critical vector for its transmission of several arboviruses, including dengue virus (DENV), Zika virus (ZIKV), chikungunya virus and filarial nematodes, such as *Dirofilaria immitis* [1–4]. Compared with *Aedes aegypti*, the primary vector for DENV and ZIKV, *Ae. albopictus* is generally considered to be a less efficient vector due to its less anthropophilic behavior and inferior adaptation to urban domestic environments [5]. However, its broad temperature adaptability and high competence for several arboviruses make it approximately as important a vector as *Ae. aegypti*. Meanwhile, combined with its strong ecological plasticity, global trade, such as the transportation of used tires and “lucky bamboo,” has also accelerated the global spread of this species from its original Southeastern Asian distribution to every continent except Antarctica [6]. Currently, it is among the top 100 worst invasive species and a threat to human health worldwide [7].

Natural environmental variation is responsible for fluctuations in insect population dynamics, distribution and biology, including population number, intensity and feeding behavior [8]. An important and rapidly adapting insect with high fecundity and a short life cycle, *Ae. albopictus* has population dynamics and vector competence that are also greatly influenced by environmental conditions [9, 10]. Numerous previous studies have discussed the effects of environmental variations on *Ae. albopictus* populations, mainly based on their influence on mosquito abundance, survival, size, fecundity and competence for certain arboviruses [11–15]. However, less research has focused on changes in the genetic diversity and population structure of *Ae. albopictus* populations associated with various environmental conditions [16–18], although this information is essential for reconstructing the dispersion patterns of *Ae. albopictus* in certain regions and providing necessary information for subsequent mosquito control.

With the advent of urbanization in China, the natural environment was altered tremendously; more people have moved from rural areas to cities, and trade has increased between cities and rural areas. All these processes create suitable habitats (e.g. waste tires, vases, plastic pails and concrete tanks) for *Ae. albopictus* and facilitate its breeding and dispersal throughout the country [19–22]. Meanwhile, the dispersion of *Ae. albopictus* from its original environment to a new environment facilitates the manifestation of new phenotypes and the increase of mutated alleles in frequency, and thriving international travel and trade provide essential routes for the continuous introduction of arboviruses from other countries, especially those in Southeast Asia, where *Ae. albopictus* originated [23], thus putting people at risk worldwide. During the past 10 years, at least 22 imported ZIKV cases were confirmed by the China CDC, and the persistent emergence of dengue in Southern China, especially in Guangdong and Yunnan provinces, is positively correlated to the widespread presence of *Ae. aegypti* and *Ae. albopictus* among these regions [24]. In contrast to *Ae. aegypti* (an invasive species distributed only in the southern area of China), *Ae. albopictus* is an indigenous Chinese mosquito, ranging from Dalian in the north to Hainan in the south [25, 26]. Moreover, it has been considered the sole agent of numerous recent dengue fever outbreaks in China [27, 28]. Its wide distribution and high competence for numerous arboviruses and nematode parasites emphasize the need to more extensively study the biology, distribution and dispersion patterns of this species.

High genetic variation is ubiquitous in vector populations, especially in invasive vectors, such as *Ae. aegypti* and *Ae. albopictus*, where it helps them occupy diverse niches and respond quickly to evolutionary challenges [29]. Microsatellites are the preferred markers for studying genetic variation in vectors due to their codominance, high informativity and vast abundance throughout vector genomes [30]. To date, multiple microsatellite loci associated with *Ae. albopictus* have been successfully isolated and employed in *Ae. albopictus* population studies on a global scale, and they continue to be a popular choice of genetic marker [15, 31–33]. Simultaneously and independently from *Ae. albopictus* microsatellites, the mitochondrial *coxI* gene has frequently been used for mosquito barcoding and invasive species monitoring because of its conservation and high accuracy when distinguishing sequence variation at the interspecies level and its usefulness in investigating population dispersal routes of mosquitoes [34–38].

To evaluate how environmental factors affect genetic diversity and dispersal patterns of *Ae. albopictus* and decipher the potential dispersal patterns of *Ae. albopictus* among different climatic regions, a total of 17 *Ae. albopictus* populations were collected and examined via 11 microsatellite loci and the *coxI* gene. The results provide information for the future control of *Ae. albopictus* in China.

## Materials and methods

### Eco-climatic characteristics of different climatic regions

All mosquitoes were sampled from the three main climatic regions of China, including tropical, subtropical and temperate regions. The annual accumulated temperature and precipitation for the tropical region is > 8000 ℃ and 1500–2000 mm, respectively, with the lowest monthly average temperature being 15–24 ℃. These data for the subtropical region are between 4500 and 8000 ℃ and > 1000 mm, respectively, with the lowest monthly average temperature being 0–15 ℃. These data for the temperate region are between 3000 and 4500 ℃ and 700 mm, respectively, with the lowest monthly average temperature being – 8 to 0 ℃ [39, 40].

#### Mosquito sampling and DNA isolation

**Fig. 1:**
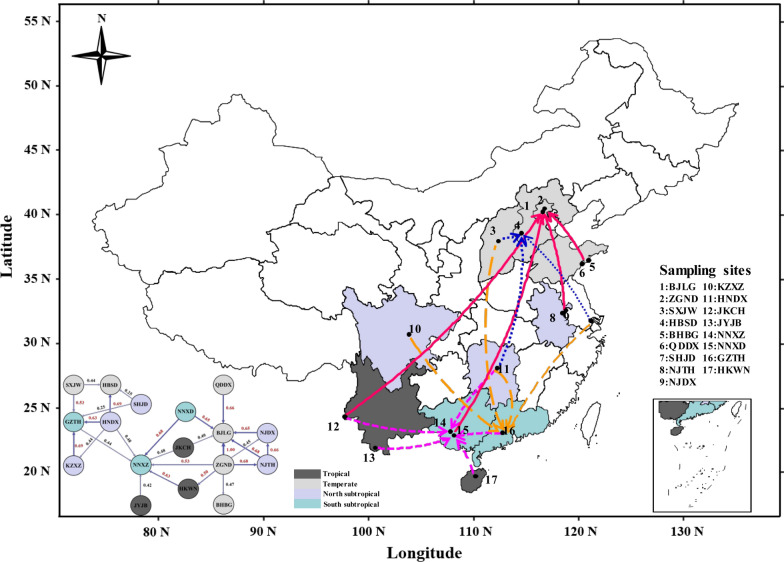
Sampling map and migration patterns for *Ae. albopictus* in three different climatic regions of China. Three climatic regions were marked with different colors, four major destinations were marked with stars of different colors, and migration routes were marked with different types of arrows

In the present study, ca. 600 *Ae. albopictus* larvae were sampled from 17 geographically diverse sites across three environmentally distinct regions of China from June to August 2018; all the sampling site information is described in Fig. 1 and Additional file 1: Table S1. To avoid inbreeding interference, each pool of larval mosquitoes for a given locality was collected from at least five wild breeding places within 500 m. For each of the samples, the larvae were reared separately and emerged at 25 ± 1 ℃ at 75 ± 5% relative humidity (RH) under a 14 h light/10 h dark (LD) photoperiod, and all female adult mosquitoes were identified under a microscope before DNA isolation [41, 42]. The DNA isolation was conducted with the Qiagen DNeasy Blood and Tissue Kit (no. 69504, Qiagen, Germany) according to the manufacturer’s protocol, and all the DNA samples were stored at − 80 °C.

#### Microsatellite isolation, processing, and coxI gene amplification

Referring to Chambers et al. [34], microsatellite markers were isolated from the whole genome of *Ae. albopictus* by magnetic-bead enrichment and PCR screening, and all markers were found to be highly polymorphic via denatured polyacrylamide gel electrophoresis (D-PAGE). A set of 11 microsatellite loci was employed for genotyping the 17 *Ae. albopictus* populations. Detailed microsatellite primer information is listed in Table 1. All PCRs were performed on a T100 Thermal Cycler (Bio-Rad, USA) in a 50 μl reaction system containing 10 ng DNA, 0.25 U PrimeSTAR HS DNA polymerase (10 pmol/µl, TaKaRa, Japan), 6 μM dNTPs (2.5 mM each, TaKaRa, Japan) and ca. 5 μM of each primer, with the program set to 35 cycles of 95 °C for 30 s, 57 °C for 30 s and 72 °C for 1 min followed by a final elongation at 72 °C for 10 min. All products were then checked with 2% agarose gel electrophoresis under UV light and run on a 3730XL DNA Genetic Analyzer (Applied Biosystems, USA).

**Table 1:** Information of microsatellite primers used for genotyping all the 17 *Ae. albopictus* populations

No.	Primer name	Forward sequence (5′–3′)	Reverse sequence (5′–3′)	Repeat motifs	Size range (bp)	Dye	GenBank no.
1	BW-P1	TTAGCATCCATCTATTCTGGC	AAACATTCCTACGCATTTCAC	(GT)6	230–260	5′-HEX	MT642446
2	BW-P3	GAAAATATGGTCTATCAAATG	AAGTCAGTAAAACAGGAGTCT	(GT)3GC(GT)3TTT	132–178	5′-FAM	MT642447
3	BW-P6	GAATTGGGAGCTTGGTAAAAC	CGCCTACTTGAGAAACACTGA	(TG)5	124–200	5′-HEX	MT642448
4	BW-P18	CACTGGTTCTCTATCGAATGC	GTGTTATCAGTTGGAAGCGTT	(GT)24	158–203	5′-HEX	MT642449
5	BW-P22	GGCGTCCCCCCAACATACATC	CAGCTCCGTCCTCCTCTTCCC	(GT)5(GCGT)2(GT)3	187–244	5′-HEX	MT642450
6	BW-P23	GGATAAGAATGACACAGGCAC	CAAAGAGGAACACCATAGGAA	(GAC)7	133–177	5′-FAM	MT642451
7	BW-P24	ACGAAACATACTTACAATTGCA	AACCTAGAGTCCGAGAGAGAAC	(AC)8	145–239	5′-FAM	MT642452
8	BW-P26	CGTGGTGGTTAGGTCCATGTT	TCGCTTTCGGCTCTAGTCAAT	(GT)5	107–233	5′-HEX	MT642453
9	BW-P27	TTATACAAAAAGCGAACATCC	CACACACATAGAAAAAAGCAA	(ACG)6	249–281	5′-FAM	MT642454
10	BW-P35	TATTTGCACATCCATTTCGTCT	TTCAAAACCTGATTTCCGACTG	(CA)7TTT	83–120	5′-HEX	MT642455
11	BW-P36	GTCATGTAGTCCTCACAGTCAC	ATATGGATCATAGATGATGGAG	(AC)6CCC	159–179	5′-FAM	MT642456

As described by Bonacum et al. [35], *coxI* sequence polymorphism was investigated among 497 individuals (22–30 individuals per locality). Briefly, DNA amplification of a 550-bp fragment of *coxI* was performed on a T100 Thermal Cycler (Bio-Rad, USA) with the following primer set: 5′-GGAGGATTTGGAAATTGATTAGTTC-3′ (F-*coxI*) and 5′-CCCGGTAAAATTAAAATATAAACTTC-3′ (R-*coxI*) in a 50 µl reaction mix containing 10 µl PCR reaction buffer (TaKaRa, Japan), 4 µl dNTPs (2.5 mM each, TaKaRa, Japan), 1 µl primers (10 pmol/µl, TaKaRa, Japan) and 0.5 µl PrimeSTAR HS DNA polymerase (TaKaRa, Japan). The PCR amplification program was set as follows: pre-denaturation at 94 °C for 3 min, followed by 35 cycles of denaturation at 94 °C for 30 s, annealing at 54 °C for 45 s and elongation at 72 °C for 1 min, with a final elongation at 72 °C for 10 min. All the PCR products were detected and separated by 2% agarose gel electrophoresis. The target fragments for *coxI* were then cut from the gel under UV light and purified with the GenElute™ PCR Clean-Up Kit (NA1020, Sigma-Aldrich, USA). Each purified PCR product was then cloned into the pCR™2.1 vector with a TA Cloning™ Kit (K202040, Invitrogen, USA) and selected by bacteria liquid PCR with the T7 promoter primers. Finally, at least 20 positive clones for each PCR product were sequenced on both strands using an ABI 3730XL automatic sequencer (Applied Biosystems, USA).

#### Population structure analyses, phylogenetic genotyping and migration analyses

The microsatellite data were processed with GeneMapper v.4.0 (Applied Biosystems, USA). All markers were tested for polymorphism by determining their polymorphism information content (PIC) values via PIC-Calc 0.6 [43], and the null allele frequency of each locus was assessed with Micro-Checker version 2.2.3 [44]. Allele frequency indices, including n_a_ and n_e_, were assessed with Cervus version 3.0.7 [45]. The Ho, He and FIS values of all populations were calculated with Arlequin version 3.5.2.2 [46]. Departure from Hardy-Weinberg equilibrium (HWE) and heterozygosity deficiency were assessed via Bottleneck version 1.2.02 [47]. Additionally, STRUCTURE version 2.3.4 [48] and the Δ*K* method of Evanno et al. [49] were employed to calculate the optimal *K* value; the parameters were set as follows: (1) *K* ranged from 1 to 20 with ten iterations for each *K* value; (2) an admixture model was chosen with a Markov chain Monte Carlo algorithm run for 100,000 iterations and 1,000,000 repetitions. The optimal *K* value was calculated via Structure Harvester: http://taylor0.biology.ucla.edu/structureHarvester/ and depicted with Distribut version 1.1. The DAPC analysis was performed on the microsatellite date of each population with the R packages “Adegenet 2.1.3” [50], and the IBD analysis was performed between genetic distance, generated from the microsatellite date, and geographical distance with “Genpop 4.7.5” [51], respectively; meanwhile, the genetic variation and F_ST_ value for each population were evaluated with the AMOVA test via Arlequin version 3.5.2.2 [46].

The haplotypes of all the populations were screened by DnaSP version 6.0 [52], and the genetic relationships among all the haplotypes were displayed as a TCS network constructed by Network 10.0.0.0 [53, 54]. Moreover, BEAST version 1.8.4 [55] and the R package “pheatmap” [56] were used to build a phylogenetic tree of all the haplotypes and examine the distribution of all the haplotypes among the different climatic regions, with the best model selected by JModelTest 2.1.10 [57]. The mismatch distribution and Bayesian skyline plot analyses were conducted with Arlequin version 3.5.2.2 and BEAST version 1.8.4, respectively, to investigate the current dispersal incidences of each population. Concurrently, the possible migration routes were rebuilt via the R package “divMigrate” with the number of bootstrap replicates set to 3, the alpha value set to 0.05, the Nm method chosen for the migration statistic and the filter threshold value set to 0.25 [58]. Finally, the relationships between the genetic indices (i.e., *n*_*e*_, Shannon index, Hd, H and *π*) and environmental factors (i.e., temperature, altitude and rainfall) were described via principal component analysis (PCA) and multiple factor analysis (MFA) with the R package “FactoMineR” [59].

## Results

### Microsatellite marker isolation and assessment

In the present study, a total of 44 pairs of microsatellite markers were isolated from the whole genome of *Ae. albopictus*, 11 pairs of which were highly polymorphic and were therefore chosen for microsatellite genotyping analysis (Table 1). The allele number of each locus ranged from 10 to 33, with a mean of 17.545 alleles per locus. The PIC values of each locus ranged from 0.334 to 0.925, with a mean value of 0.713. According to the definition of PIC values by Allah et al. [60], nearly all the markers selected were highly informative (PIC value > 0.5); the exception was BW-P18, which had a value of 0.357 and was considered to be an informative marker. The Micro-Checker results suggested that null alleles were present at all loci, with frequencies ranging from 0.064 to 0.157, with an average of 0.078 (Additional file 2: Table S5); however, frequency values < 0.2 are considered to have no significant effect on the accuracy of data analysis by many studies [61, 62]. The linkage disequilibrium (LD) test showed that 302 pairs of loci of a total of 1870 (16.15%) across all locations were in significant LD after Bonferroni correction, while no consistency was found among them (Additional file 3: Figure S1).

#### Genetic diversity and variation

According to the results of the microsatellite data analysis, the observed number of alleles (n_a_) in each *Ae. albopictus* population was very high, and the mean n_a_ value in each climatic region ranged from 6.909 to 8.091 without a significant difference. In contrast, the effective number of alleles (*n*_*e*_) ranged from 3.501 to 4.525, and the n_e_ value increased from the temperate region (3.876) to the tropical region (4.144). The mean value of observed heterozygosity (Ho) for all the climatic regions was ca. 0.557, which was significantly lower than the expected heterozygosity (He) (ca. 0.684). The F_IS_ value of each climatic region ranged from 0.266 to 0.359, and the *Ae. albopictus* populations significantly departed from HWE, except in two locations, SHJD and KZXZ (subtropical region, Table 2). Heterozygosity tests of all 17 *Ae. albopictus* populations based on the stepwise mutation model (SMM) revealed that nearly all the populations from the temperate and subtropical regions displayed significant population expansion with *p* values < 0.05, while no significance was observed among all the tests for the *Ae. albopictus* populations from the tropical region after Bonferroni correction (Additional file 4: Table S2).

**Table 2:** Genetic diversity indices for 17 *Ae. albopictus* populations investigated by 11 microsatellite loci

Regions	SC	SS	n_a_	n_e_	F_IS_	*Ho*	*He*	HWE
Tropical region	HKWN	30	8.455	4.525	0.388	0.561	0.733	0.442^***^
	JYJB	30	7.636	4.355	0.385	0.584	0.699	0.535^***^
	JKCH	25	7.000	3.553	0.292	0.532	0.671	0.246^***^
	Mean	29	7.697	4.144	0.355	0.559	0.701	0.408^***^
South-subtropical region	NNXD	30	7.909	4.000	0.336	0.533	0.700	0.347^**^
	NNXZ	30	7.909	4.190	0.473	0.627	0.725	0.480^***^
	GZTH	30	8.455	4.292	0.172	0.467	0.714	0.192^*^
	Mean	30	8.091	4.161	0.327	0.542	0.713	0.340^**^
North-subtropical region	NJTH	30	7.455	3.836	0.392	0.590	0.682	0.473^***^
	NJDX	30	8.455	4.119	0.465	0.637	0.706	0.551^***^
	KZXZ	30	6.182	3.681	0.189	0.474	0.677	0.331
	SHJD	30	5.455	3.106	0.035	0.455	0.606	0.123
	Mean	30	6.909	3.677	0.266	0.542	0.660	0.369^*^
Temperate region	HNDX	30	7.000	3.642	0.247	0.553	0.627	0.365^**^
	QDDX	30	7.909	3.921	0.361	0.570	0.664	0.380^**^
	BHBG	22	8.091	3.838	0.433	0.641	0.637	0.447^***^
	BJLG	30	8.636	4.341	0.408	0.574	0.728	0.456^***^
	ZGND	30	6.909	3.728	0.425	0.631	0.636	0.419^***^
	SXJW	30	6.455	3.501	0.270	0.572	0.622	0.421^**^
	HBSD	30	7.546	3.926	0.256	0.508	0.687	0.341^**^
	Mean	29	7.591	3.876	0.359	0.583	0.662	0.411^**^

*SC* sample code, *SS* sample size, *n*_*a*_ observed number of alleles, *n*_*e*_ effective number of alleles (Kimura and Crow 1964), *HWE* Hardy-Weinberg disequilibrium, *Ho* observed heterozygosity, *He* expected heterozygosity (Nei’s 1973)

^***^p < 0.001

^**^p < 0.01

^*^p < 0.05

In all, a total of 25 *coxI* haplotypes were observed among 497 *Ae. albopictus* individuals (GenBank ID: MN651301-MN651325) based on an analysis of the *coxI* sequences. The haplotype indices (i.e., Hd, H, and *π*) changed dramatically across *Ae. albopictus* populations from different climatic regions (Additional file 5: Table S3). The haplotype diversity (Hd) ranged from 0.074 (ZGND, temperate region) to 0.750 (JYJB, tropical region), with the nucleotide diversity (*π*) ranging from 0.014 (BJLG, temperate region) to 0.190 (JYJB, tropical region). The average number of nucleotide differences (*k*) ranged from 0.067 (BJLG, temperate region) to 0.929 (JYJB, tropical region), and the number of polymorphic sites ranged from 1 to 5 across all the populations. Overall, the tropical *Ae. albopictus* populations showed the highest diversity, with mean values of Hd, *π* and *k* reaching 0.657, 0.156 and 0.763, respectively, whereas the temperate populations showed the lowest diversity.

#### Population structure and differentiation based on microsatellite analysis

**Fig. 2:**
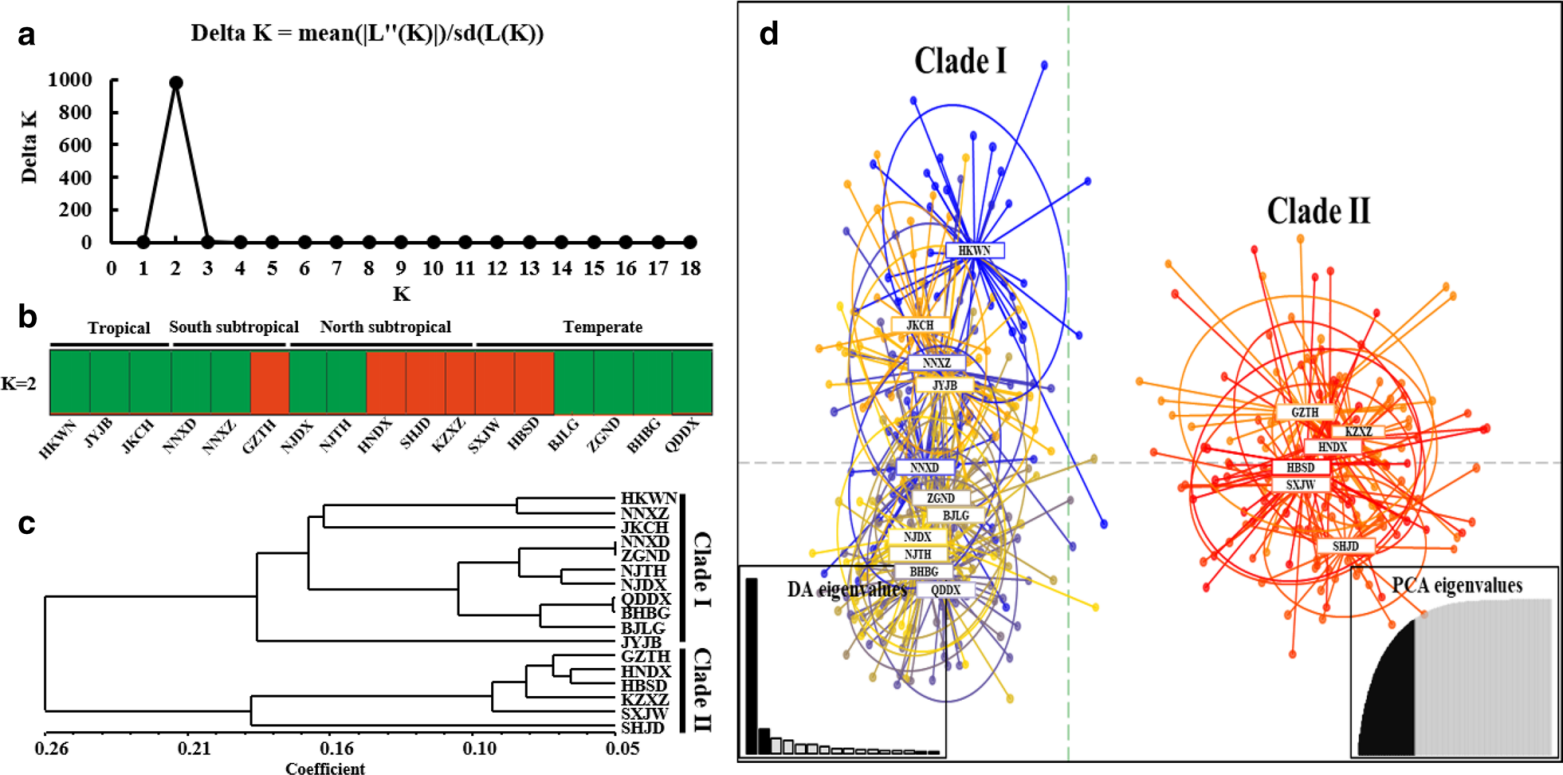
Population structure analysis of all 17 *Ae. albopictus* populations based on 11 microsatellite loci. **a** K values assessed via Evanno et al.’s ΔK methods; **b** STRUCTURE bar plots (*k* = 2); **c** Bayesian clustering analysis of all *Ae. albopictus* populations; **d** DAPC analysis of all *Ae. albopictus* populations, and 86.4% of variation was explained by 50 PCs

In the present study, according to the microsatellite analysis results, all the *Ae. albopictus* populations were adequately allocated to two clades with significant genetic differences, and the best *K* value, as determined via the Δ*K* method of Evanno et al., was also equal to two (Fig. 2a). Combined with the STRUCTURE bar plot analysis, the Bayesian clustering analysis showed that all the *Ae. albopictus* populations were adequately allocated to two clades with certain locations from the subtropical and temperate regions genetically isolated from the other locations. (Fig. 2b and c). Moreover, a total of 86.4% of the variation was explained by 50 PCs in the discriminant analysis of principal components (DAPC) analysis; the results revealed two genetically isolated groups, and there was no clear relationship between the *Ae. albopictus* population structure and its distribution across climatic regions (Fig.2d).

The AMOVA results are presented in Table 3. Of the total genetic variation partitioned, 31.40% could be attributed to differences among individuals within populations and 63.04% to differences within individuals (*F*_IS_ = 0.33253, *F*_IT_ = 0.36962, and all *p* < 0.00001). Meanwhile, the pairwise *F*_ST_ values between populations ranged from 0.008 (ZGND, temperate region and NNXD, subtropical region) to 0.141 (SHJD, subtropical region and JKCH, tropical region). Genetic differentiation was significant between all the sampled populations after Bonferroni correction (*p* < 0.05) except for five pairs of F_ST_ values among the subtropical populations NNXZ, NJTH, NJDX, KZXZ and temperate populations ZGND, BHBG, QDDX and HBSD (Additional file 6: Table S4). In contrast to the high individual variation, a slightly significant positive correlation was observed among only the temperate populations via isolation by distance (IBD) analysis (*R*^2^ = 0.6614, *p* = 0.048), while no such evidence was observed in the other two regions (Additional file 7: Figure S2).

**Table 3:** Analysis of molecular variance (AMOVA) test of 17 *Ae. albopictus* populations sampled from three climatic regions.

Source of variation	d. f.	Sum of squares	Variance components	Percentage of variation	*P *value	Fixation indices
Among groups	5	142.425	0.12789Va	3.62	*P* < 0.00001	F_CT_ = 0.03620
Among populations within groups	11	93.157	0.06845Vb	1.94	*P* < 0.00001	F_SC_ = 0.02010
Among individuals within populations	485	2156.367	1.10951Vc	31.40	*P* < 0.00001	F_IS_ = 0.33253
Within individuals	502	1118.000	2.22709Vd	63.04	*P* < 0.00001	F_IT_ = 0.36962
Total	1003	3509.949	3.53294			

#### Haplotype network and phylogenetic analysis based on coxI sequences

**Fig. 3:**
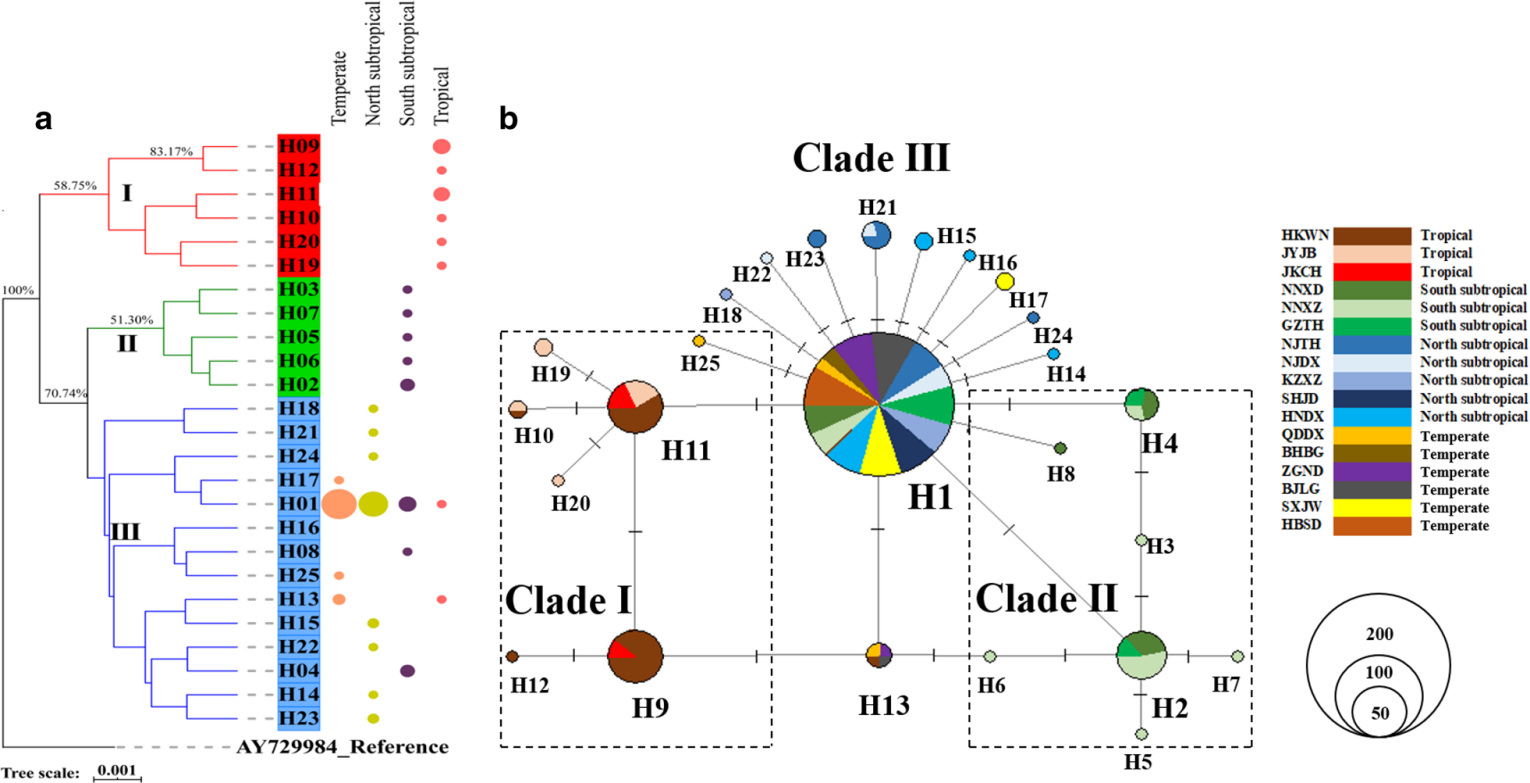
Bayesian and haplotype network analysis of the representing 25 haplotypes among all 17 *Ae. albopictus* populations based on *coxI* sequences. **a** Bayesian tree of the representing 25 haplotypes was constructed via BEAST version 1.8.4 with the best model “HKY+G” selected by JModelTest 2.1.10; **b** TCS haplotype network for the *coxI* gene of all *Ae. albopictus* individuals (*n* = 502) from different climatic regions of China. The sizes of circles are proportional to haplotype frequency, and each line segment represents a single mutation

Three major haplotype clades, distributed across the three central climatic regions and closely related to each other, were reconstructed via the TCS network with 497 *coxI* sequences. Haplotype I (H1) of Clade III was the most frequent haplotype and was distributed from the tropical to the temperate region with an increasing trend. Nearly all the other haplotypes derived from H1 with one or two mutations, with Clade I mainly distributed in the tropical region and Clade II mainly distributed in the southern subtropical region (Fig. 3b).

A phylogenetic tree of all 25 haplotype sequences, combined with a heatmap analysis, demonstrated that the 25 haplotypes were divided into three major well-supported clades. As expected, Clade I was separated from Clade II and Clade III with 100% bootstrap support and included haplotypes H9, H10, H11, H12, H19 and H20, which were observed in only the tropical region. In comparison, Clade II diverged from Clade III with lower bootstrap support (70.74%) and included five haplotypes (H2–H3 and H5–H7) that were distributed in only the southern subtropical region. Clade III was an admixture group containing all the remaining 14 haplotypes, 2 of which were observed in the tropical region and four in the climatic regions (Fig. 3a). Meanwhile, the Tajima’s D and Fu’s F values were negative, with no statistically significant *p* values except in population JKCH (tropical region, Additional file 5: Table S3). Based on the *coxI* sequences, the mismatch analysis results showed that the Harpending raggedness indices for all three haplotype clades were relatively low (ranging from 0.1054 to 0.1812, *p* > 0.05), and unimodal mismatch distributions were observed among the different *Ae. albopictus* populations (Additional file 8: Figure S3).

#### Migration and correlation analyses between genetic indices and environmental factors

**Fig. 4:**
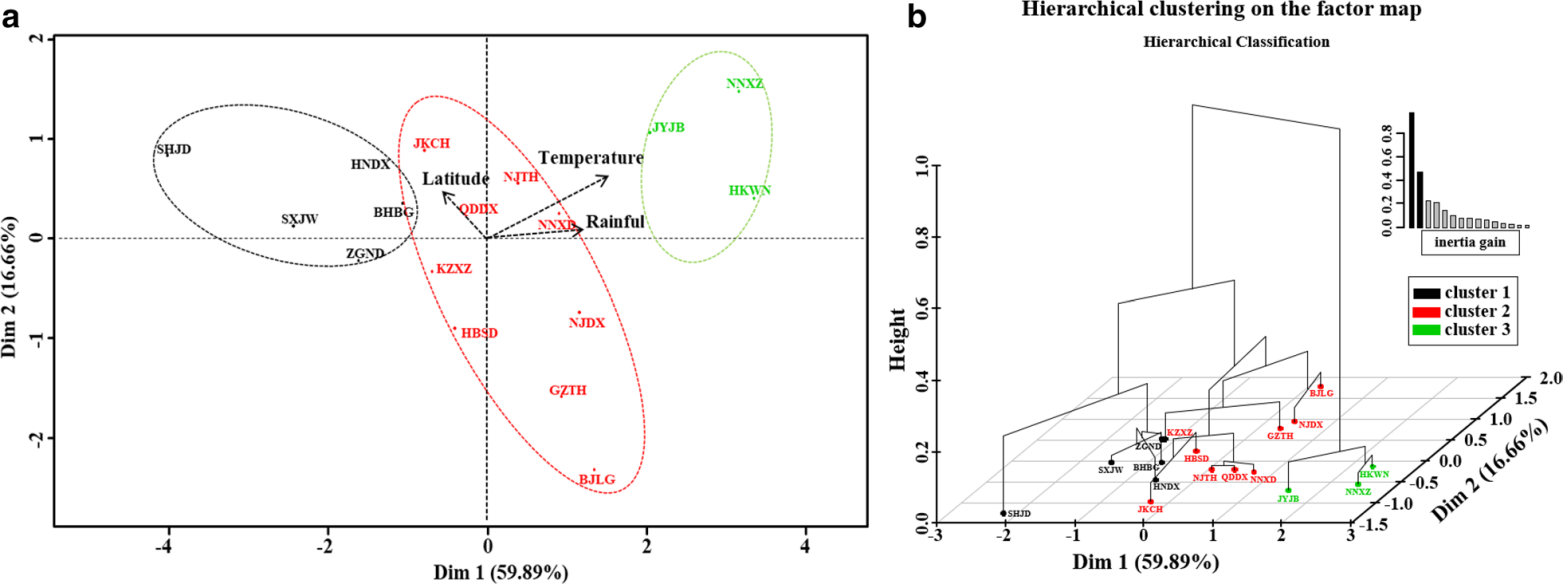
Multiple factor analysis (MFA) and hierarchical clustering performed on 17 *Ae. albopictus* populations sampled from three different climatic regions. **a** Multiple factor analysis between genetic indices of all individuals and three environmental variables (temperature, rainfall and altitude) of 17 *Ae. albopictus* populations, populations of different clusters were marked with different colors; **b** hierarchical clustering of all 17 *Ae. albopictus* populations on the factor map

As illustrated in Fig. 4, all five genetic indices (i.e., *n*_*e*_, Shannon index, Hd, *H* and *π*) and two environmental factors (i.e., temperature and rainfall) contributed equivalently to the first axis of the PCA, explaining up to 95.2% of the variation; the exception was the environmental factor latitude, which contributed more to the second axis, with a proportion of 17.5%. Combining these results with hierarchical clustering via MFA, all 17 *Ae. albopictus* populations were clustered into three groups, of which cluster II and cluster III were closely related to each other. When environmental factors were regarded as the major influencing factors, the molecular diversity indices (i.e., Hd, *k* and *π*) of the populations in the tropical region were significantly higher than those in the other regions (Additional file 5: Table S3).

Migration patterns were assessed using divMigrate networks representing all 17 *Ae. albopictus* populations. As expected, *Ae. albopictus* was observed to have migrated frequently between the tropical and temperate areas in both directions (Fig. 1). A total of four major migration trends were observed among the different climatic regions with high gene flow (Nm > 0.4). Two trends were found to have originated from the tropical and subtropical areas and reached Hebei and Beijing in the temperate area, while another two arrived in Guangdong and Guangxi from the southern subtropical area. Compared with the south to north routes, the latter migration routes were substantially larger.

## Discussion

Due to their high mutation rates, codominant expression and universal distribution throughout the eukaryotic genome, microsatellite loci have been widely used to evaluate the genetic variation and population structure of mosquitoes, especially for those without fully annotated genomes [32, 34]. Although different sets of microsatellite loci have been identified by previous studies [31, 32, 63], the absence of PCR amplification products of mosquito samples from certain climatic regions has resulted in insufficient data. Therefore, in this study, we developed new primers for our samples to ensure the integrity of the experimental data. However, the potential impact of null alleles, such as reducing population genetic diversity and increasing genetic differentiation among populations [61, 64, 65], must be taken into consideration when microsatellite markers are used, because null alleles are more frequent in arthropods than in other species [66–68]. Mitochondrial genes are also good markers for studying population diversity and are widely used in many studies. To improve the accuracy of the findings in this study, the mitochondrial *coxI* gene was also employed, and the genetic variation of all the individuals was investigated using both *coxI* and microsatellite data.

Consistent with the studies of Zhong et al. [69] and Wei et al. [70], which were based on only mitochondrial and microsatellite diversity, respectively, the tropical *Ae. albopictus* populations showed higher diversity than the populations of the other two regions in China. An environment more conducive to survival and continuous dispersion may be the best explanation for this phenomenon. According to studies by Wei et al. and others [70–73], the hot and humid climate of Southern China is more suitable for the breeding and development of *Ae. albopictus* than a temperate climate, which has relatively low temperatures and dry air. Notably, during the winter, freezing temperatures bring about diapause in *Ae. albopictus* for the whole winter period, resulting in lower allele richness and population diversity in temperate *Ae. albopictus* populations [66–68]. Meanwhile, Southern China shares borders with many countries in Southeast Asia, where *Ae. albopictus* originates geographically, including Laos, the Philippines, and Myanmar [23]. Frequent border trades and resident travel among these areas result in the continuous dispersion of *Ae. albopictus*, enriching the diversity of the local population; this result was confirmed by the results of the bottleneck analysis, which showed that all the populations from temperate and subtropical regions had undergone bottleneck effects, while the populations of the tropical region had not. As suggested by Takezaki et al. [74], an He value between 0.5 and 0.8 indicates high genetic polymorphism in the population. In the present study, the He values of all the sampling sites ranged from 0.606 to 0.733, which indicates high polymorphism. Simultaneously, compared with those in the tropical region, the mean Ho values of the temperate and subtropical regions were significantly lower than the mean He values, and nearly all the populations significantly departed from HWE. This phenomenon may also be closely related to the less suitable climate and the need to develop diapause during wintertime among populations of these regions as mentioned before, and our present sampling method (larvae rather than adults) may also be the reason for the significant departure from HWE of all populations. Meanwhile, the continuous dispersion of *Ae. albopictus* from Southeast Asian countries to the tropical region of China also augments the local mosquito population (Additional file 9: Figure S4).

In the present study, nearly 44% (11 out of 25) of these 25 detected haplotypes were observed only in the tropical and subtropical regions. The same result for Chinese *Ae. albopictus* populations was also observed by both Guo et al. and Zhang et al. [16, 75]. Guo et al. identified three predominant haplotypes in *Ae. albopictus* populations: H01 (21.5%) from Guangdong Province (subtropical region), H19 (22.1%) from Yunnan (tropical region) and Hainan provinces (tropical region), and H30 (10%) from Hainan Province (tropical region); likewise, Zhang et al. also detected 25 haplotypes across China, and 48% (12 out of 25) of these haplotypes were detected in only tropical and subtropical regions. Both studies corroborate our findings that the haplotype diversity of the tropical and subtropical regions is higher than that of the temperate region. Continuous dispersal from neighboring Southeast Asian countries may be the reason for the higher diversity of the *Ae. albopictus* populations in the tropical and subtropical regions and that the origins of the *Ae. albopictus* populations of these two regions may be different. Furthermore, the mismatch analysis results revealed recent population dispersal among the different climatic regions, which is the main reason for the universal distribution of the remaining haplotypes. As an important and rapidly adapting insect, *Ae. albopictus* has biological characteristics that are strongly influenced by environmental conditions [9, 10]. In tropical and subtropical regions, the climate is hot and wet, making it more suitable for the survival of *Ae. albopictus*; when this mosquito migrates from a tropical region to a temperate region, it needs time to adapt to the new environment, especially to survive during the winter period, which causes diapause in *Ae. albopictus* [4]. According to a study by Roiz et al. about the effects of environmental factors on *Ae. albopictus*, the activity of host-seeking females was positively affected by temperature and rainfall [76]. The PCA in the present study examined genetic indices and environmental factors (such as temperature, rainfall and altitude) and revealed that temperature and rainfall may be the leading causes of differences in genetic diversity among *Ae. albopictus* populations from different climatic regions.

Corresponding to the results of Kotsakiozi and Manni et al. [32, 77], which showed no genetic differentiation within the native Asian range, our population differentiation analysis results showed that even though nearly all the pairwise *F*_ST_ values between populations were significant, the values were not high, which indicates potential anthropogenic exchange between the populations. A study by Schmidt et al. [78] of the genetic structure of *Ae. albopictus* collected from 12 sample sites in Guangzhou also found that human transportation networks, particularly shipping terminals, influenced the genetic structure of *Ae. albopictus* populations. With the development of China, the rapid expansion of high-speed rail, aircraft routes and highways between different climatic regions has not only facilitated trade among different cities but also accelerated the spread of mosquitoes. Accordingly, only two genetically separated clades were observed among these populations, and molecular variations within populations and individuals were found to contribute to the genetic differentiation between these populations. As a global invasive species, *Ae. albopictus* has developed several strategies to cope with a broad range of temperatures and adapt to local thermal conditions [9, 71], which helps this mosquito disperse and colonize different locations successfully. Here, the migration analysis results displayed four major migration trends with high gene flow (Nm > 0.4) among the different climatic regions, corresponding with China’s main trade routes (e.g. pan-Pearl River Delta and Bohai Rim Business District, etc.) and tourism routes (e.g. Beijing-Guangzhou railway, Beijing-Shanghai railway and Beijing-Kowloon railway). All these results revealed that human-aided dispersion might be the main reason for the similarity of populations across the different regions [25, 70–74, 77–83]. This hypothesis was also verified via IBD analysis, which indicated that long distances were not significantly associated with individual genetic variation, except among individuals from the tropical region with a significant *p* value of the Mantel test.

Overall, strong dispersal patterns were observed in Southern China, and frequent dispersals have occurred from the tropical and subtropical regions to the temperate region. Monitoring the population structure and potential migration routes of *Ae. albopictus* in different climatic regions will help in the selection of appropriate mosquito control strategies, and since dengue fever continues to break out in Southern China every year, monitoring the potential migration routes of *Ae. albopictus* can also predict the potential transmission routes of this virus and help prevent its further spread.

## Conclusion

The present study systematically evaluated the genetic variation, population structure and haplotype-based phylogenetic relationships of *Ae. albopictus* populations across different climatic regions of China. All 17 *Ae. albopictus* populations were genetically structured into two isolated clades, in accordance with their locality or region of origin, and three major haplotype clusters were observed via phylogenetic analysis of *coxI*. These results suggest different evolutionary histories in changing environments, especially differences in temperature and rainfall. Meanwhile, four major migration trends were observed among the different climatic regions with high gene flow; these trends contribute to the similarity of the *Ae. albopictus* populations.

## Supplementary Information

**Additional file 1: Table S1.** Sampling information of 17 *Ae. albopictus* populations collected from three different climatic regions of China.

**Additional file 2: Table S5.** Genetic diversity of 11 microsatellite loci developed for *Ae. albopictus* based on samples (*n* = 502) collected from three different climatic regions of China.

**Additional file 3: Figure S1.** Linkage disequilibrium analysis at each pair of Loci across all 17 *Ae. albopictus* populations.

**Additional file 4: Table S2.** Heterozygosity tests of all 17 *Ae. albopictus* populations based on SMM model.

**Additional file 5: Table S3.** Haplotype diversity of 17 *Ae. albopictus* populations based on *coxI* collected from three different climatic regions of China.

**Additional file 6: Table S4.** Population differentiation estimation of the F_ST_ value (below the diagonal) and Geographic distance (above the diagonal) among all 17 *Ae. albopictus* populations.

**Additional file 7: Figure S2.** Isolation by distance analysis of all 17 *Ae. albopictus* populations.

**Additional file 8: Figure S3.** Historical demography analysis of *Ae. albopictus* inferred from mtDNA *coxI* sequences.

**Additional file 9: Figure S4.** Migration analysis of all 17 *Ae. albopictus* populations inferred from microsatellite data.

## Data Availability

Data are available on request to the corresponding author.

## Abbreviations

*coxI*: Mitochondrial cytochrome c oxidase subunit 1
PIC: Polymorphism Information Content
Ho: Observed heterozygosity
He: Excepted heterozygosity
HWE test: Hardy-Weinberg equilibrium test
AMOVA test: Analysis of molecular variation test
IBD: Isolation by distance
WHO: World Health Organization
CDC: Centers for Disease Control
n_a_: Observed number of alleles
n_e_: Effective number of alleles
F_IS_: Inbreeding coefficient
F_ST_: Genetic differences among populations
F_IT_: Genetic differences within individuals
PCA: Principal component analysis
MFA: Multiple factor analysis
LD: Linkage disequilibrium
Hd: Haplotype diversity
DAPC: Discriminant analysis of principal components
Nm: Number of migrants
