# Supplementary material for: Dispersal patterns and population genetic structure of Aedes albopictus (Diptera: Culicidae) in three different climatic regions of China
Source: Parasit Vectors. 2021 Jan 6;14:12. doi: 10.1186/s13071-020-04521-4 (PMC7789686; doi:10.1186/s13071-020-04521-4)
Supplement: Supplementary file 1 — Additional file 1: Table S1. Sampling information of 17 Ae. albopictus populations collected from three different climatic regions of China. [file 13071_2020_4521_MOESM1_ESM.doc]

**1. Additional File 1：TableS1 Sampling information of 17 *Aedes albopictus* populations collected from three different temperature zones of China.**

| No. | Code | Province | Temperature zone | Geographical coordinate | Sample size | Temperature | Rainfall | Altitude | Date of collection |
| --- | --- | --- | --- | --- | --- | --- | --- | --- | --- |
| 1 | HKWN | Haikou | Tropical | 20°00′30.85″N,110°21′48.21″E | 30 | 27~34℃ | 846 mm | 25 m | 06/10/2018 |
| 2 | JYJB | Yunnan | Tropical | 22°00'44.35''N,100°49'15.04''E | 30 | 23~32℃ | 569 mm | 555 m | 07/12/2018 |
| 3 | JKCH | Yunnan | Tropical | 23°58′59.77″N, 97°52′13.70″E | 25 | 22~30℃ | 794 mm | 867 m | 06/25/2018 |
| 4 | NNXD | Guangxi | South Subtropical | 22°50′22.91″N,108°12′50.30″E | 30 | 25~32℃ | 605 mm | 135 m | 07/18/2018 |
| 5 | NNXZ | Guangxi | South Subtropical | 22°49′03.12″N,108°20′53.49″E | 30 | 25~32℃ | 605 mm | 99 m | 07/22/2018 |
| 6 | GZTH | Guangzhou | South Subtropical | 22°50′22.91″N,108°12′50.30″E | 30 | 25~32℃ | 916 mm | 5 m | 08/01/2018 |
| 7 | NJTH | Jiangsu | North Subtropical | 32°04′14.62″N,118°45′55.74″E | 30 | 23~31℃ | 660 mm | 6 m | 06/06/2018 |
| 8 | NJDX | Jiangsu | North Subtropical | 32°03′25.02″N,118°46′27.42″E | 30 | 23~31℃ | 660 mm | 8 m | 06/08/2018 |
| 9 | KZXZ | Sichuan | North Subtropical | 23°08′28.79″N,113°25′20.27″E | 30 | 22~30℃ | 597 mm | 504 m | 08/23/2018 |
| 10 | SHJD | Shanghai | North Subtropical | 30°39′58.42″N,104°03′04.69″E | 30 | 25~31℃ | 570 mm | 10 m | 06/01/2018 |
| 11 | HNDX | Hunan | North Subtropical | 37°41'09.24''N,112°41'45.99''E | 30 | 24~32℃ | 474 mm | 31 m | 07/30/2018 |
| 12 | QDDX | Shandong | Temperate | 36°04′15.87″N,120°25′19.49″E | 30 | 22~27℃ | 340 mm | 46 m | 08/20/2018 |
| 13 | BHBG | Shandong | Temperate | 36°03′57.48″N,120°22′40.33″E | 22 | 22~27℃ | 340 mm | 26 m | 08/18/2018 |
| 14 | BJLG | Beijing | Temperate | 39°57′47.38″N,116°18′15.34″E | 30 | 22~30℃ | 399 mm | 59 m | 08/06/2018 |
| 15 | ZGND | Beijing | Temperate | 40°00′13.09″N,116°21′03.44″E | 30 | 22~30℃ | 399 mm | 55 m | 08/06/2018 |
| 16 | SXJW | Shanxi | Temperate | 31°13′43.70″N,121°29′16.27″E | 30 | 18~29℃ | 270 mm | 806 m | 08/30/2018 |
| 17 | HBSD | Hebei | Temperate | 28°10'51.16''N,112°56′21.72″E | 30 | 23~31℃ | 372 mm | 71 m | 08/28/2018 |

No.: Number
