# Supplementary material for: Dispersal patterns and population genetic structure of Aedes albopictus (Diptera: Culicidae) in three different climatic regions of China
Source: Parasit Vectors. 2021 Jan 6;14:12. doi: 10.1186/s13071-020-04521-4 (PMC7789686; doi:10.1186/s13071-020-04521-4)
Supplement: Supplementary file 2 — Additional file 2: Table S5. Genetic diversity of 11 microsatellite loci developed for Ae. albopictus based on samples (n = 502) collected from three different climatic regions of China. [file 13071_2020_4521_MOESM2_ESM.doc]

**Additional File 2 TableS2** Heterozygosity tests of all 17 *Ae. albopictus* populations based on SMM model.

| **Subtropical Region** | | NNXD | NNXZ | GZTH | NJTH | NJDX | KZXZ | SHJD | HNDX |
| --- | --- | --- | --- | --- | --- | --- | --- | --- | --- |
| SMM | *He < Heq* | 10 | 8 | 10 | 8 | 10 | 7 | 8 | 8 |
|  | *He > Heq* | 1 | 3 | 1 | 3 | 1 | 4 | 3 | 3 |
|  | *P (He < Heq)* | 0.001^***^ | 0.037^*^ | 0.001^***^ | 0.034^*^ | 0.001^***^ | 0.110 | 0.033^*^ | 0.033^*^ |

| **Temperate Region** | | QDDX | BHBG | BJLG | ZGND | SXJW | HBSD |
| --- | --- | --- | --- | --- | --- | --- | --- |
| SMM | *He < Heq* | 9 | 10 | 10 | 9 | 7 | 8 |
|  | *He > Heq* | 2 | 1 | 1 | 2 | 4 | 3 |
|  | *P (He < Heq)* | 0.008^**^ | 0.001^***^ | 0.001^***^ | 0.007^**^ | 0.116 | 0.033^*^ |

| **Tropical Region** | | HKWN | JYJB | JKCH |
| --- | --- | --- | --- | --- |
| SMM | *He < Heq* | 6 | 6 | 7 |
|  | *He > Heq* | 5 | 5 | 4 |
|  | *P (He < Heq)* | 0.264 | 0.275 | 0.121 |

Number of loci exhibiting heterozygosity excess (*He*) and expected heterozygosity based on the number of observed alleles (*Heq*) under the SMM model. Significant *P*-values are marked.

^***^: *p*<0.001; ^**^: *p*<0.01; ^*^: *p*<0.05

SMM model: Stepwise Mutation Model (Cornuet and Luikart, 1996)
