# Supplementary material for: Dispersal patterns and population genetic structure of Aedes albopictus (Diptera: Culicidae) in three different climatic regions of China
Source: Parasit Vectors. 2021 Jan 6;14:12. doi: 10.1186/s13071-020-04521-4 (PMC7789686; doi:10.1186/s13071-020-04521-4)
Supplement: Supplementary file 3 — Additional file 3: Figure S1. Linkage disequilibrium analysis at each pair of Loci across all 17 Ae. albopictus populations. [file 13071_2020_4521_MOESM3_ESM.doc]

**Additional File 3 TableS3 Haplotype diversity of 17 *Ae. albopictus* populations based on COI collected from three different climatic regions of China.**

| No. | SS | Region | N | H | Hd | π | k | S | Tajima's *D* | Fu's *Fs* | Haplotypes |
| --- | --- | --- | --- | --- | --- | --- | --- | --- | --- | --- | --- |
| 1 | Tropical | JKCH | 5 | 2 | 0.600 | 0.122 | 0.600 | 1 | 1.225 | 0.626 | H9(2), H11(3) |
| 2 | Tropical | JYJB | 8 | 4 | 0.750 | 0.190 | 0.929 | 3 | -0.812 | -1.387 | H10(1), H11(4), H19(2), H20(1) |
| 3 | Tropical | HKWN | 30 | 6 | 0.621 | 0.155 | 0.759 | 4 | -0.638 | -2.509 | H9(16), H1(1), H10(10), H11(10), H12(1), H13(1) |
| 4 | South subtropical | NNXZ | 30 | 7 | 0.658 | 0.180 | 0.883 | 5 | -0.82 | -3.204 | H1(16), H2(8), H3(1), H4(2), H5(1), H6(1), H7(1) |
| 5 | South subtropical | NNXD | 30 | 4 | 0.487 | 0.110 | 0.540 | 3 | -0.674 | -1.035 | H1(21), H4(3), H8(1), H7(5) |
| 6 | South subtropical | GZTH | 30 | 3 | 0.248 | 0.053 | 0.257 | 2 | -1.004 | -1.174 | H1(26), H2(2), H3(2) |
| 7 | North subtropical | NJDX | 17 | 3 | 0.228 | 0.048 | 0.235 | 2 | -1.504 | -1.68 | H1(15), H21(1), H22(1) |
| 8 | North subtropical | NJTH | 30 | 4 | 0.402 | 0.089 | 0.434 | 3 | -1.003 | -1.53 | H1(23), H21(4), H23(2), H24(1) |
| 9 | North subtropical | HNDX | 28 | 4 | 0.267 | 0.057 | 0.280 | 3 | -1.527 | -2.61 | H1(24), H14(1), H15(2), H4(1) |
| 10 | North subtropical | SHJD | 25 | 1 | na | na | na | na | na | na | H1(25) |
| 11 | North subtropical | KZXZ | 22 | 2 | 0.091 | 0.019 | 0.091 | 1 | -1.162 | -0.957 | H1(21), H18(1) |
| 12 | Temperate | BJLG | 30 | 2 | 0.067 | 0.014 | 0.067 | 1 | -1.147 | -1.211 | H1(30), H13(1) |
| 13 | Temperate | ZGND | 27 | 2 | 0.074 | 0.015 | 0.074 | 1 | -1.154 | -1.125 | H1(26), H13(1) |
| 14 | Temperate | BHBG | 30 | 1 | na | na | na | na | na | na | H1(10) |
| 15 | Temperate | QDDX | 9 | 3 | 0.417 | 0.091 | 0.444 | 2 | -1.362 | -1.081 | H1(7), H13(1), H25(1) |
| 16 | Temperate | HBSD | 27 | 1 | na | na | na | na | na | na | H1(27) |
| 17 | Temperate | SXJW | 29 | 2 | 0.133 | 0.027 | 0.133 | 1 | -0.753 | -0.41 | H1(27), H17(2) |

No.: Number; SS: Sampling Size; N: number of sequences; H: number of haplotypes; Hd: Haplotype diversity; π: nucleotide diversity; k: average number of nucleotide differences; S: number of polymorphic sites; na: not applicable. PIC: Polymorphic Information Content;

Tajima's *D* & Fu's *Fs*, Not significant, *P*>0.10
