# Supplementary material for: Dispersal patterns and population genetic structure of Aedes albopictus (Diptera: Culicidae) in three different climatic regions of China
Source: Parasit Vectors. 2021 Jan 6;14:12. doi: 10.1186/s13071-020-04521-4 (PMC7789686; doi:10.1186/s13071-020-04521-4)
Supplement: Supplementary file 4 — Additional file 4: Table S2. Heterozygosity tests of all 17 Ae. albopictus populations based on SMM model. [file 13071_2020_4521_MOESM4_ESM.doc]

**Additional File 4 TableS4** Population differentiation estimation of the *Fst* value (below the diagonal) and Geographic distance (Km, above the diagonal) between all 17 Ae. albopictus populations.

| **pop** | **HKWN** | **NNXZ** | **JKCH** | **NNXD** | **ZGND** | **BJLG** | **NJTH** | **NJDX** | **QDDX** | **BHBG** | **JYJB** | **GZTH** | **KZXZ** | **HNDX** | **SXJW** | **HBSD** | **SHJD** |
| --- | --- | --- | --- | --- | --- | --- | --- | --- | --- | --- | --- | --- | --- | --- | --- | --- | --- |
| **HKWN** | **-** | 375.540 | 1361.447 | 385.428 | 2295.793 | 2290.354 | 1580.829 | 1580.023 | 2037.943 | 2035.385 | 1015.401 | 470.521 | 1343.479 | 945.357 | 1978.825 | 2040.268 | 1671.301 |
| **NNXZ** | **0.019** | **-** | 1077.222 | 13.982 | 2054.580 | 2048.842 | 1452.953 | 1452.530 | 1877.985 | 1874.923 | 779.156 | 520.824 | 971.290 | 753.485 | 1705.044 | 1787.733 | 1600.678 |
| **JKCH** | **0.055** | **0.048** | **-** | 1063.243 | 2482.691 | 2476.731 | 2234.754 | 2235.056 | 2544.154 | 2540.325 | 373.190 | 1587.639 | 961.550 | 1574.834 | 2074.936 | 2217.558 | 2457.092 |
| **NNXD** | **0.041** | **0.016** | **0.037** | **-** | 2056.941 | 2051.190 | 1460.520 | 1460.111 | 1884.036 | 1880.957 | 765.677 | 534.336 | 963.286 | 759.849 | 1705.828 | 1789.652 | 1609.940 |
| **ZGND** | **0.052** | **0.035** | **0.040** | *0.008* | **-** | 6.021 | 908.659 | 910.340 | 564.100 | 562.117 | 2480.894 | 1895.534 | 1521.820 | 1351.877 | 408.145 | 273.823 | 1080.323 |
| **BJLG** | **0.052** | **0.036** | **0.045** | **0.018** | **0.021** | **-** | 905.310 | 906.992 | 563.288 | 561.268 | 2474.873 | 1890.446 | 1515.878 | 1346.519 | 402.226 | 267.830 | 1078.128 |
| **NJTH** | **0.058** | **0.041** | **0.068** | **0.024** | *0.017* | **0.025** | **-** | 1.742 | 470.478 | 468.635 | 2095.549 | 1123.419 | 1404.886 | 707.729 | 834.229 | 764.231 | 274.096 |
| **NJDX** | **0.061** | **0.041** | **0.053** | **0.023** | **0.030** | **0.019** | *0.012* | **-** | 471.671 | 469.837 | 2095.568 | 1122.487 | 1405.648 | 707.495 | 835.936 | 765.972 | 272.811 |
| **QDDX** | **0.073** | **0.056** | **0.082** | **0.037** | **0.026** | **0.025** | **0.030** | **0.032** | **-** | 4.015 | 2456.373 | 1588.037 | 1632.426 | 1124.750 | 709.921 | 566.211 | 547.622 |
| **BHBG** | **0.075** | **0.052** | **0.080** | **0.044** | **0.024** | **0.034** | **0.033** | **0.038** | *0.014* | **-** | 2452.766 | 1585.728 | 1628.459 | 1121.724 | 706.288 | 562.816 | 547.812 |
| **JYJB** | **0.044** | **0.055** | **0.054** | **0.031** | **0.048** | **0.056** | **0.070** | **0.062** | **0.071** | **0.084** | **-** | 1299.977 | 1014.612 | 1399.024 | 2081.864 | 2208.169 | 2290.747 |
| **GZTH** | **0.036** | **0.029** | **0.066** | **0.022** | **0.044** | **0.035** | **0.042** | **0.033** | **0.047** | **0.070** | **0.056** | **-** | 1249.636 | 562.343 | 1619.105 | 1655.525 | 1201.256 |
| **KZXZ** | **0.056** | **0.062** | **0.101** | **0.057** | **0.080** | **0.068** | **0.074** | **0.065** | **0.072** | **0.086** | **0.070** | **0.022** | **-** | 904.108 | 1113.800 | 1258.962 | 1662.525 |
| **HNDX** | **0.058** | **0.054** | **0.117** | **0.051** | **0.063** | **0.073** | **0.053** | **0.064** | **0.064** | **0.088** | **0.068** | **0.029** | **0.024** | **-** | 1057.609 | 1101.767 | 892.140 |
| **SXJW** | **0.070** | **0.071** | **0.116** | **0.058** | **0.069** | **0.063** | **0.048** | **0.056** | **0.060** | **0.088** | **0.080** | **0.033** | **0.041** | **0.031** | **-** | 163.378 | 1078.586 |
| **HBSD** | **0.062** | **0.050** | **0.086** | **0.042** | **0.053** | **0.049** | **0.044** | **0.046** | **0.050** | **0.071** | **0.064** | **0.018** | *0.016* | **0.019** | **0.026** | **-** | 986.205 |
| **SHJD** | **0.105** | **0.090** | **0.141** | **0.074** | **0.095** | **0.091** | **0.092** | **0.103** | **0.083** | **0.103** | **0.111** | **0.076** | **0.084** | **0.085** | **0.095** | **0.066** | **-** |

The significances were tested for multi comparisons by the Bonferroni method, all *p*-values less than 0.05 were considered significant and given in bold, and *p*-values more than 0.05 were given in italic.
