# Supplementary figures and images for: Dispersal patterns and population genetic structure of Aedes albopictus (Diptera: Culicidae) in three different climatic regions of China
Source: Parasit Vectors. 2021 Jan 6;14:12. doi: 10.1186/s13071-020-04521-4 (PMC7789686; doi:10.1186/s13071-020-04521-4)

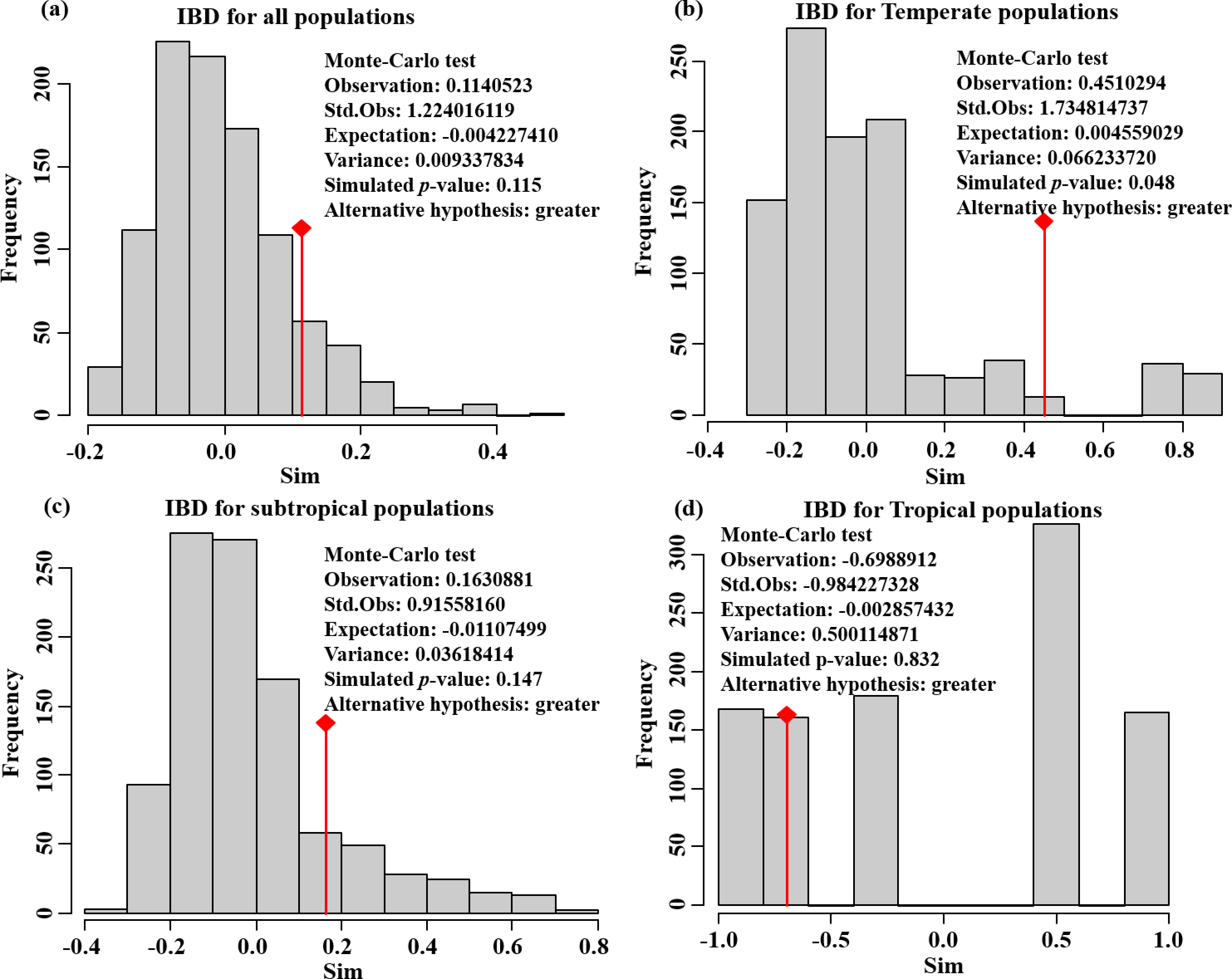

Supplement: Supplementary file 6 — Additional file 6: Table S4. Population differentiation estimation of the FST value (below the diagonal) and Geographic distance (above the diagonal) among all 17 Ae. albopictus populations. [file 13071_2020_4521_MOESM6_ESM.tif]

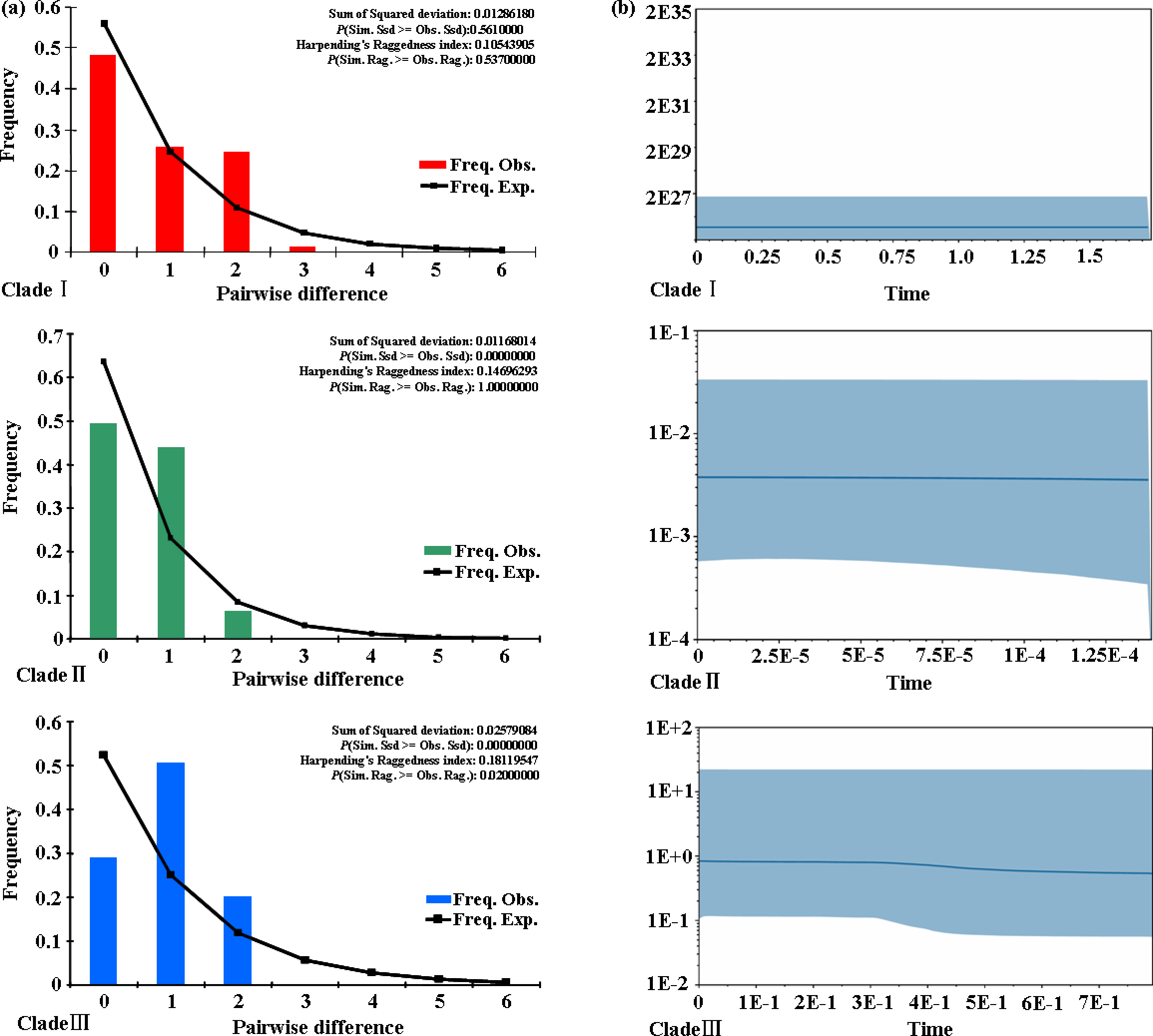

Supplement: Supplementary file 7 — Additional file 7: Figure S2. Isolation by distance analysis of all 17 Ae. albopictus populations. [file 13071_2020_4521_MOESM7_ESM.tif]

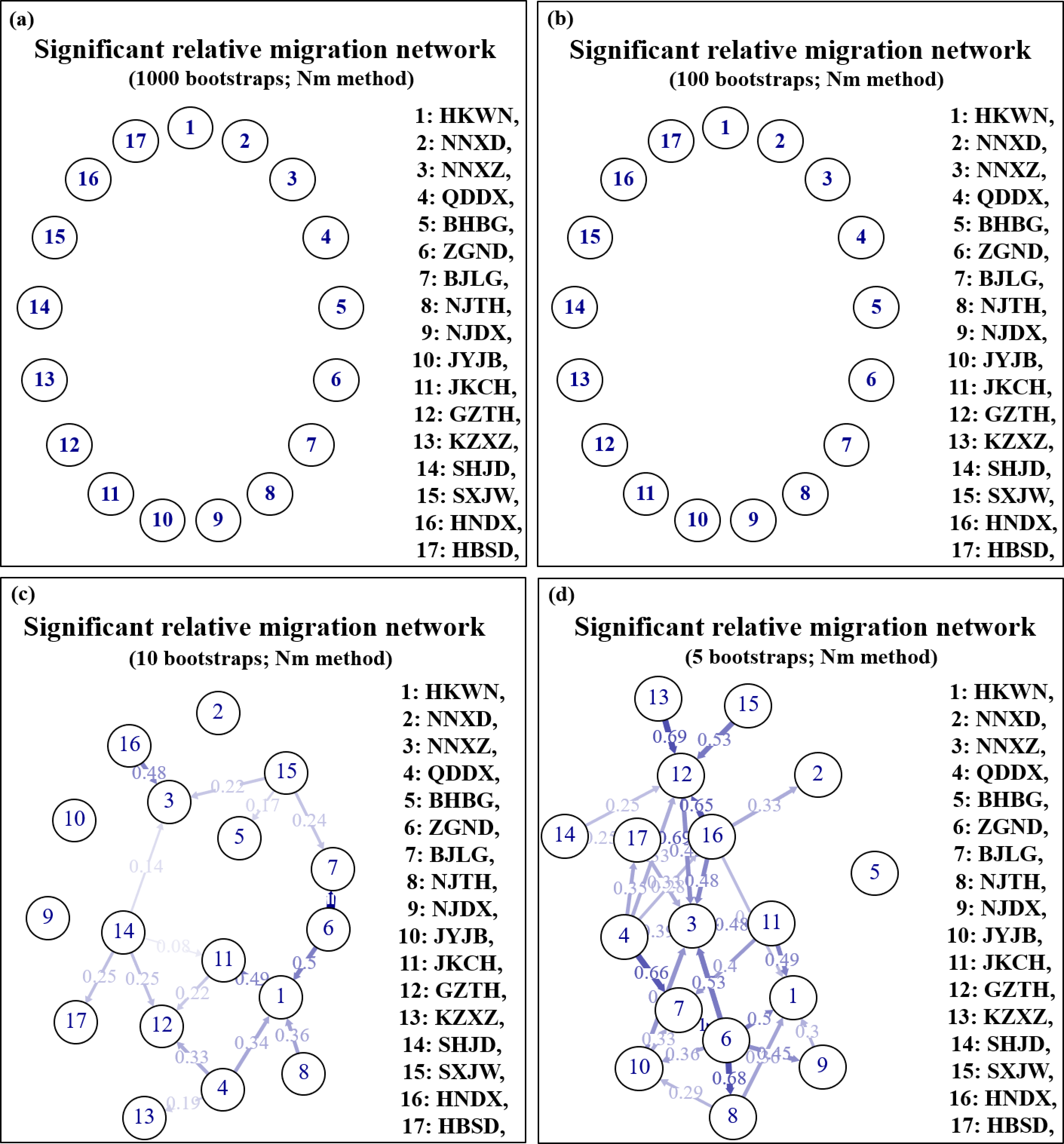

Supplement: Supplementary file 9 — Additional file 9: Figure S4. Migration analysis of all 17 Ae. albopictus populations inferred from microsatellite data. [file 13071_2020_4521_MOESM9_ESM.tif]
