# Supplementary material for: Dispersal patterns and population genetic structure of Aedes albopictus (Diptera: Culicidae) in three different climatic regions of China
Source: Parasit Vectors. 2021 Jan 6;14:12. doi: 10.1186/s13071-020-04521-4 (PMC7789686; doi:10.1186/s13071-020-04521-4)
Supplement: Supplementary file 8 — Additional file 8: Figure S3. Historical demography analysis of Ae. albopictus inferred from mtDNA coxI sequences. [file 13071_2020_4521_MOESM8_ESM.doc]

**Additional File 8 TableS5 Genetic diversity of 11 microsatellite loci developed for *Ae. albopictus* based on samples(n=502) collected from three different climatic regions of China.**

| **No.** | **Maker name** | **Sample Number** | **Allele number** | **Gene Diversity** | **PIC** | **Null Allele Frequency** |
| --- | --- | --- | --- | --- | --- | --- |
| 1 | BW-P1 | 496 | 15 | 0.608 | 0.736 | 0.018 |
| 2 | BW-P3 | 497 | 10 | 0.807 | 0.526 | 0.142 |
| 3 | BW-P6 | 502 | 18 | 0.779 | 0.579 | 0.045 |
| 4 | BW-P18 | 502 | 14 | 0.925 | 0.357 | 0.089 |
| 5 | BW-P22 | 502 | 28 | 0.398 | 0.860 | 0.020 |
| 6 | BW-P23 | 501 | 21 | 0.370 | 0.876 | 0.064 |
| 7 | BW-P24 | 497 | 33 | 0.334 | 0.891 | 0.124 |
| 8 | BW-P26 | 498 | 19 | 0.528 | 0.790 | 0.050 |
| 9 | BW-P27 | 463 | 12 | 0.520 | 0.802 | 0.157 |
| 10 | BW-P35 | 495 | 13 | 0.519 | 0.800 | 0.011 |
| 11 | BW-P36 | 480 | 10 | 0.739 | 0.623 | 0.141 |
|  | **Mean** | **494** | **17.545** | **0.593** | **0.713** | **0.078** |

No.: Number

PIC: Polymorphic Information Content
